# Complex post-transcriptional regulation of EGF-receptor expression by EGF and TGF-α in human prostate cancer cells

**DOI:** 10.1038/sj.bjc.6690407

**Published:** 1999-05

**Authors:** D Seth, K Shaw, J Jazayeri, P J Leedman

**Affiliations:** Laboratory for Cancer Medicine and University Department of Medicine, Royal Perth Hospital, Perth, Western Australia, 6001, Australia

**Keywords:** prostate cancer, EGF-receptor, mRNA stability, protein stability, autocrine loop

## Abstract

The epidermal growth factor receptor (EGFR) plays an important role in the development and progression of prostate cancer and its overexpression is associated with decreased survival. With progression, prostate cancer cells switch from epidermal growth factor (EGF) to transforming growth factor α (TGF-α) synthesis, which contributes to autocrine growth and unrestrained proliferation. To define the molecular mechanisms involved in the regulation of EGFR expression by EGF and TGF-α we studied three human prostate cancer cell lines, androgen-responsive (LNCaP) and -unresponsive (DU145 and PC3). Here we show that TGF-α stabilized EGFR mRNA two- to threefold in all three cell lines, whilst EGF stabilized EGFR mRNA ∼ twofold in LNCaP and DU145 cells, but not in PC3 cells. Both ligands increased EGFR transcription in LNCaP and DU145 cells, with less effect in PC3 cells. In all three cell lines EGF reduced total EGFR protein levels more than TGF-α, but this was associated with a greater increase in de novo protein synthesis with EGF compared to TGF-α. Only EGF, however, shortened EGFR protein stability (half-life decreased from 5 h to 120 min), resulting in rapid disappearance of newly synthesized EGFR protein. Both ligands increased total LNCaP and DU145 cell numbers. These studies demonstrate that the EGF- and TGF-α-induced upregulation of EGFR mRNA and protein in human prostate cancer cell lines is complex and occurs at multiple, transcriptional and post-transcriptional levels. Taken together, these data provide novel insight into the molecular mechanisms by which TGF-α would preferentially maintain an autocrine loop in human prostate cancer cells. Furthermore, this work suggests that in human prostate cancer cells ligand-specific differential intracellular trafficking of the EGFR plays a major role in regulating its expression. © 1999 Cancer Research Campaign


*British Journal of Cancer* (1999) **80**(5/6), 657–669
© 1999 Cancer Research Campaign
Article no. bjoc.1998.0407

### Complex post-transcriptional regulation of EGF-receptor expression by EGF and TGF-α in human prostate cancer cells

**D Seth, K Shaw, J Jazayeri and PJ Leedman**

Laboratory for Cancer Medicine and University Department of Medicine, Royal Perth Hospital, Box X2213 GPO, Perth, Western Australia 6001, Australia

**Summary** The epidermal growth factor receptor (EGFR) plays an important role in the development and progression of prostate cancer and its overexpression is associated with decreased survival. With progression, prostate cancer cells switch from epidermal growth factor (EGF) to transforming growth factor α (TGF-α) synthesis, which contributes to autocrine growth and unrestrained proliferation. To define the molecular mechanisms involved in the regulation of EGFR expression by EGF and TGF-α we studied three human prostate cancer cell lines, androgen-responsive (LNCaP) and -unresponsive (DU145 and PC3). Here we show that TGF-α stabilized EGFR mRNA two- to threefold in all three cell lines, whilst EGF stabilized EGFR mRNA ~ twofold in LNCaP and DU145 cells, but not in PC3 cells. Both ligands increased EGFR transcription in LNCaP and DU145 cells, with less effect in PC3 cells. In all three cell lines EGF reduced total EGFR protein levels more than TGF-α, but this was associated with a greater increase in de novo protein synthesis with EGF compared to TGF-α. Only EGF, however, shortened EGFR protein stability (half-life decreased from 5 h to 120 min), resulting in rapid disappearance of newly synthesized EGFR protein. Both ligands increased total LNCaP and DU145 cell numbers. These studies demonstrate that the EGF- and TGF-α-induced upregulation of EGFR mRNA and protein in human prostate cancer cell lines is complex and occurs at multiple, transcriptional and post-transcriptional levels. Taken together, these data provide novel insight into the molecular mechanisms by which TGF-α would preferentially maintain an autocrine loop in human prostate cancer cells. Furthermore, this work suggests that in human prostate cancer cells ligand-specific differential intracellular trafficking of the EGFR plays a major role in regulating its expression.

**Keywords:** prostate cancer; EGF-receptor; mRNA stability; protein stability; autocrine loop

Prostate cancer remains the most frequently diagnosed solid tumour and is the second leading cause of cancer-related death in men in Western countries (Nomura and Kolonel, 1991). Most prostate cancers are treated with androgen ablation, resulting in subjective improvement in ~ 70%. However, the response is usually temporary and offers no realistic possibility of cure (Byrne et al, 1996). Current data suggest that transformed prostate cells are able to overcome normal growth restraints by producing growth factors, such as epidermal growth factor (EGF) and transforming growth factor α (TGF-α), which act through autocrine and paracrine mechanisms to stimulate growth (Wilding et al, 1989; Xie et al, 1995).

The epidermal growth factor receptor (EGFR) and two of its multiple high affinity ligands, EGF and TGF-α, play an important role in the development of several different human cancers (Todaro et al, 1979; Lippman, 1993), including prostate cancer (Ching et al, 1993). Several studies have shown that prostate cancer cells express EGF, TGF-α and EGFR mRNA and protein (Morris and Dodd, 1990; Ching et al, 1993; Glynne-Jones et al, 1996). Further, recent immunohistochemical and in situ hybridization analysis showed that the levels of EGFR and TGF-α mRNA and protein are increased in carcinoma cells compared to benign epithelium (Ching et al, 1993; Glynne-Jones et al, 1996). Overexpression of the EGFR is also associated with a worse clinical prognosis (Gullick, 1991; Modjtahedi and Dean, 1994). To date, all prostate cancer cell lines tested (including DU145, PC3, LNCaP, ALVA101 and ARCaP) express increased EGFRs (Liu et al, 1993; Glynne-Jones et al, 1996; Zhau et al, 1996). The latter cell line is highly invasive and metastatic, and has greatly increased EGFRs (Zhau et al, 1996). Most of these cell lines are growth-stimulated by EGF and TGF-α in culture (Davies and Eaton, 1989; Ching et al, 1993), and growth-inhibited by EGFR antibodies (Ennis et al, 1989; Mendelsohn, 1992). Moreover, recent data indicate that chimeric monoclonal EGFR antibodies can significantly inhibit the growth of DU145 and PC3 cell xenografts in nude mice (Prewett et al, 1997). Analysis of the responses to EGF and TGF-α in the androgen-independent DU145 and PC3 cell lines which overexpress EGFRs suggests that part of the progression to hormone-independence involves a ‘switch’ in secretion from EGF to TGF-α (Ching et al, 1993), and development of an autocrine loop. Evidence supporting the existence of an autocrine loop involving TGF-α and the EGFR has been obtained in prostate carcinoma specimens (Glynne-Jones et al, 1996), as well as in prostate cancer cell lines (Ching et al, 1993; Liu et al, 1993). In addition, secretion of TGF-α changes from paracrine to autocrine in late-stage disease, which may contribute to the refractory nature of these tumours to hormonal therapy (Connolly and Rose, 1990, 1991). Taken together, these data indicate that the

*Received 14 May 1998*
*Revised 6 October 1998*
*Accepted 28 October 1998*

*Correspondence to:* PJ Leedman

EGF/TGF-α–EGFR pathway serves as a key growth regulator in prostate cancer.

Relatively little is known about the molecular mechanisms involved in the regulation of EGFR mRNA and protein expression by EGFR and TGF-α, and their complex relationship with androgen in prostate cancer cells. A decrease in EGFR binding sites has been observed with EGF in some studies, but the mechanism of the decrease has not been fully elucidated (Hanover et al, 1985; Turkeri et al, 1994). Modulation of EGFR expression by androgens is complex. Androgens have been shown to up-regulate (Schuurmans et al, 1988, 1991) or down-regulate (Traish and Wotiz, 1987; Connolly and Rose, 1990) EGFR expression. For example, dihydrotestosterone (DHT) has been shown to up-regulate EGFR mRNA ~ two-fold in androgen-responsive ALVA101 prostate cancer cells (Liu et al, 1993). In studies using the PC3 cell line stably transfected with the androgen receptor (AR), DHT increased EGFR mRNA and protein expression ~ two-fold (Brass et al, 1995). In non-prostate human carcinoma cells there is considerable evidence for regulation of EGFR expression at the post-transcriptional level: (i) EGF increases EGFR mRNA in breast MDA-468 and epidermoid KB cells by increasing the stability of the mRNA (Jinno et al, 1988) and (ii) thyroid hormone dramatically reduces EGFR mRNA stability in A431 cells (Kesavan et al, 1991). The possibility that stabilization of EGFR mRNA may play an important role in the development of the TGF-α-EGFR autocrine loop in prostate cancer cells has not been explored.

To better define the molecular mechanisms involved in regulation of EGFR expression by EGF and TGF-α and the role of the EGFR pathway in prostate cancer cell growth, we studied three human prostate cancer cell lines (LNCaP, DU145 and PC3). Here we show that EGF and TGF-α differentially regulate EGFR mRNA and protein synthesis, and that the regulation is exerted at transcriptional and multiple post-transcriptional levels. At the mRNA level, the predominant effect of TGF-α is to stabilize EGFR mRNA, whilst EGF predominantly increases EGFR transcription. Interestingly, TGF-α reduces the stability of EGFR protein less than EGF. This supports a central role for TGF-α in the 'switch' that promotes development and maintenance of the autocrine TGF-α–EGFR loop in these cells. Both ligands induced proliferation of LNCaP and DU145 cells; however, TGF-α induced significantly more proliferation than EGF at 24 h in LNCaP cells. Thus, the TGF-α–EGFR pathway plays an important role in the proliferative response in androgen-responsive and -unresponsive prostate cancer cells. These data define novel differences in the molecular mechanisms of action of EGF and TGF-α in human prostate cancer cells, and provide a molecular mechanism for the preferential role of TGF-α in the maintenance of the autocrine loop. Furthermore, they suggest that ligand -specific differential intracellular trafficking plays a major role in regulating EGFR expression in these cells.

### MATERIALS AND METHODS

### Cell lines and reagents

Cell lines were obtained from ATCC (Rockville, MD, USA). LNCaP, DU145 and PC3 cells were grown and maintained in RPMI-1640 (minus phenol red) media supplemented with 10% non-heat-inactivated fetal calf serum (NHI-FCS) (Gibco-BRL, Sydney, Australia) and 1% Pen/Strep (100 U ml$^{-1}$ penicillin, 100 mg ml$^{-1}$ streptomycin) at 37°C and 5% carbon dioxide. For experiments, cells were grown to 70% confluence in 60 mm dishes, serum starved for 24 h and the effect of EGF (0.25–16 nM), TGF-α (0.25–16 nM) (Promega Corp., Madison, WI, USA), DHT (10 nM) and cycloheximide (CHX) (10 μg ml$^{-1}$) (Sigma, St Louis, MO, USA) on EGFR expression was determined.

### Cell proliferation assays

Equal numbers of cells were plated in triplicate (~1 × 10$^4$ per well), serum starved for 24 h and exposed to EGF or TGF-α (4 nM) and/or DHT (10 nM) for various time intervals. The cells were harvested by trypsinizing in 100-μl phosphate-buffered

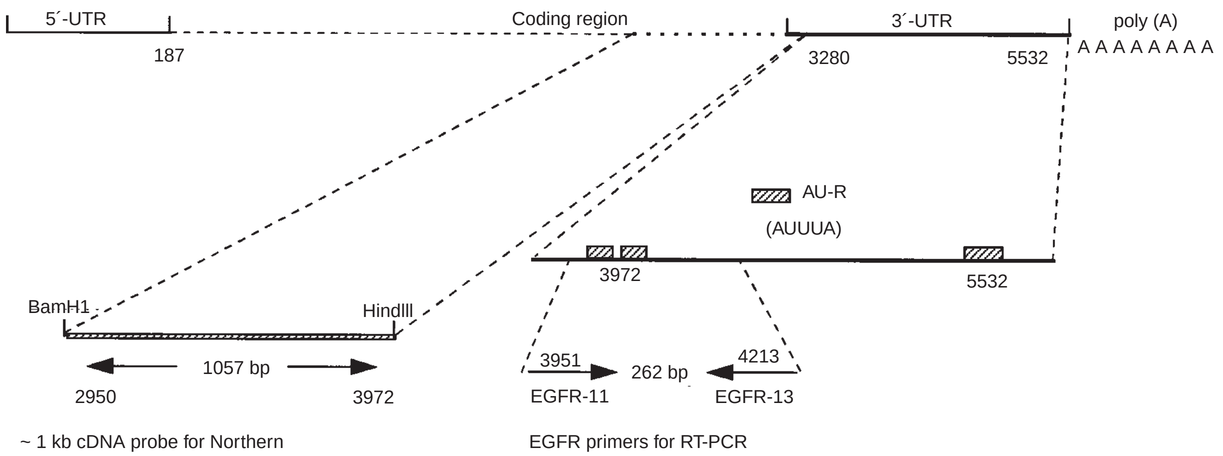

**Figure 1** Schematic of EGFR mRNA. Line diagram of EGFR mRNA depicting 5′-UTR, coding region, 3′-UTR and poly(A) tail. For multiplex PCR the primers EGFR-11 (3951) and EGFR-13 (4213) in the AU-rich region (AU-R) of the 3′-UTR were used. An ~ 1 kb probe (2950–3972) from the EGFR coding region was used in the Northern analysis. Also shown are the positions of the AU-R regions (AUUUA) in the 3′-UTR of EGFR mRNA

© Cancer Research Campaign 1999

saline (PBS) and cell numbers counted using a haemocytometer under 100 × magnification of a Leitz Wetzlar microscope.

### cDNA probes

An ~ 1 kb *Bam*H1/Hin*d*III fragment containing the 3′ end of the coding region and 131 bp from the 3′-untranslated region (3′-UTR) of the EGFR cDNA (see Figure 1) was random primed using $^{32}$P dCTP (3000 Ci mmol$^{-1}$, Amersham, Australia) and used to probe for EGFR mRNA. A 1.1 kb *Eco*R1/*Bam*H1 fragment from a plasmid encoding rat 18S ribosomal RNA was labelled with $^{32}$P dCTP and used as a probe for normalization.

### RNA isolation and Northern analysis

Cells were lysed in 4 M guanidinium isothiocyanate (GTC) (ICN) and total RNA isolated using phenol–chloroform extraction. RNA (10–15 μg) was size fractionated on a 1% agarose–formaldehyde gel, transferred and UV cross-linked to Hybond-N+ membrane (Amersham, Australia), prehybridized and hybridized in formamide buffer overnight at 42°C with a $^{32}$P dCTP labelled EGFR cDNA probe with at least $10^6$ cpm ml$^{-1}$. The membrane was washed and analysed by autoradiography using Kodak EM-1 film at −80°C and quantitated using ImageQuant software (Molecular Dynamics). For normalization, the membranes were stripped by boiling in 1% sodium dodecyl sulphate (SDS) for 10 min and reprobed with a rat 18S ribosomal RNA cDNA probe.

### mRNA turnover studies

Cells were grown to 70% confluence in 60-mm dishes in standard media, serum starved for 24 h and treated with EGF, TGF-α or CHX for 8 h (controls were untreated) followed by the addition of the transcription inhibitor actinomycin D (5 μg ml$^{-1}$, Sigma, St Louis, MO, USA). Total RNA was isolated from the cells at 0, 2, 4, 6, 8 and 12 h time intervals and subjected to Northern analysis or multiplex reverse transcription polymerase chain reaction (RT-PCR) (described below). mRNA half-life was determined using linear regression analysis.

### Reverse transcription

Total RNA (1–4 μg) was reverse transcribed with oligo dT using AMV-reverse transcriptase (Promega Corp., Madison, WI, USA) at 42°C for 1 h. The first strand cDNA was stored at 4°C and used as template for subsequent multiplex PCR.

### Oligonucleotides for multiplex PCR

EGFR primers were designed from the 3′UTR of EGFR flanking the AU-rich region (Figure 1) which amplified a 262 bp fragment. EGFR-11 (3951–3985): 5′-GACTAGATCTCCACCGAG-GATAGTATGAGCCCTA; EGFR-13 (4213–4183): 5′-CTAGA-GATCTAAGCTTCTTCCTTGTTGGAA. The primers used for normalizing were either from human β-actin cDNA generating an amplicon of 202 bp – β-actin sense (1298–1318): 5′-GCCAA-CACAGTGCTGTCTGG; β-actin antisense (1500–1481): 5′-TACTCCTGCTTGCTGATCCA – or GAPDH primers from a human cDNA lung cancer cell amplifying 309 bp fragment on PCR – GAPDH sense (60–88): 5′-GTGAAGGTCGGAGT-CAACG; GAPDH antisense (369–349): 5′-GGTGAA-GACGCCAGTGGACTC. Oligonucleotides were obtained from Biotech International Ltd, Australia. Multiplex PCR was performed at 94°C denaturing, 58°C annealing and 72°C extension for 35 cycles in a Corbett FTS 320, Australia, thermal cycler using 50–100 ng each primer for 25 μl reaction volume. The bands were resolved on a 3.5% agarose (ScientifiX, Australia) gel and quantitated using ImageQuant software.

### Nuclear run-on transcription assay

Exponentially growing cells were treated with EGF or TGF-α for 6–8 h. Cells were washed twice with PBS and trypsinized. Nuclei were isolated in the presence of NP40, centrifuged briefly, resuspended in nuclear storage buffer (50 mM Tris–HCl, pH 8.0, 40% glycerol, 0.1 mM EDTA, 0.1 mM DTT) and stored in Nunc vials in liquid nitrogen. For the transcription assay, the method used was as described (Ausubel et al, 1994). Membranes were analysed by PhosphorImager and quantitated using ImageQuant software.

### Anti-phosphotyrosine ECL Western

This was performed as previously described (Tilbrook et al, 1996). Briefly, cells were serum starved for 24 h, exposed to 1 nM, 4 nM and 16 nM EGF or TGF-α for different (0–60 min) time periods, washed and lysed in anti-phosphotyrosine lysis buffer and the lysate incubated with 2 μg sheep polyclonal anti-human EGFR antibody (Upstate Biotechnology, USA). After overnight adsorption onto protein A beads the eluate was subjected to 7% SDS-PAGE and immunoblotted prior to incubation with a 1:1000 dilution of antiphosphotyrosine IgG2bk (Upstate Biotechnology, USA) followed by a 1:5000 dilution of anti-mouse HRP antibody. Protein was visualized by enhanced chemiluminescence (ECL; Amersham, UK) and bands quantitated using a Kodak Digital DCS-420c camera and ImageQuant software. To normalize for protein loading on the gel, membranes were incubated with primary and secondary antibodies exactly as described below for the EGFR Western blot assay.

### Western blot assay

Cells were serum starved for 24 h and treated with EGF or TGF-α for 6–8 h, harvested and lysed in ice-cold lysis buffer (1% Triton X-100, 20 mM Tris–HCI pH 7.4, 1 mM EDTA). The lysate was incubated for 10 min on ice, centrifuged at 1500 ***g*** for 10 min at 4°C, and the supernatant snap-frozen and stored at −80°C. Total protein concentrations were determined using the Bio-Rad protein assay kit. Protein (15–20 μg per lane) was electrophoresed on 7% SDS-PAGE gels and transferred to nitrocellulose membrane. The membrane was blocked with 5% skim milk in 1 × TBS-T (20 mM Tris–HCl pH 7.4, 150 mM sodium chloride, 0.1% Tween-20) at 4°C overnight, incubated with a 1:2000 dilution of a sheep polyclonal anti-human EGFR antibody (Upstate Biotechnology, Santa Cruz, CA, USA) for 1 h at room temperature, and then washed and incubated with horseradish peroxidase-conjugated anti-sheep goat IgG antibody (1:2000 dilution) (Amersham, UK). The protein was visualized and bands quantitated as above.

### Metabolic labelling of cells

Cells were serum starved for 24 h and exposed to 4 nM EGF or TGF-α for 6 h. For immunoprecipitation experiments, media were

© Cancer Research Campaign 1999 

replaced with methionine-free EMEM media (Cytosystems, Australia) containing 2% FCS for 2 h and labelled for 1 h ± EGF or TGF-α with 200 μCi of Promix L$^{35}$S-methionine/cysteine (Amersham, Australia) as described (Beguinot et al, 1984). Briefly, cells were washed, lysed in lysis buffer and the lysate ($2 \times 10^7$ cpm) was incubated with 3 μg sheep polyclonal anti-human EGFR antibody (Upstate Biotechnology, USA) for 2 h at 4°C and the immunoprecipitate adsorbed onto protein A Sepharose beads. Beads were washed and the eluate subjected to 7% SDS-PAGE. To estimate the rate of EGFR protein synthesis, cells were treated as above except that EGFR protein was isolated at various time points after addition of the radioisotope. For protein decay assays, the L$^{35}$S-methionine/cysteine was replaced with media ± EGF or TGF-α after 1 h, and EGFR protein determined at different time intervals, thereafter.

### Statistical analysis

Statistical analysis was performed using the Student's unpaired *t*-test and results are shown at the 0.05% level of significance.

### RESULTS

### EGF and TGF-α up-regulate EGFR mRNA in androgen-dependent and -independent prostate cancer cell lines

In preliminary experiments we evaluated the biological potency of each ligand by determining the concentration of EGF and TGF-α that produced comparable levels of phosphotyrosine activation of the EGFR in each cell line. Incubation of LNCaP and DU145 cells with 25 ng ml$^{-1}$ (4 nM) EGF and 20 ng ml$^{-1}$ (4 nM) TGF-α respectively, induced an equivalent level of EGFR tyrosine phosphorylation at 15 and 30 min time points (Figure 2). Thus, for subsequent experiments we used 4 nM EGF and 4 nM TGF-α unless otherwise specified. To determine the effect of dose–response for each ligand on EGFR mRNA, cells were incubated with either EGF (0.25–16 nM), TGF-α (0.25–16 nM) or CHX (10 μg ml$^{-1}$) for various time intervals, total RNA extracted and analysed by either Northern hybridization or semi-quantitative multiplex RT-PCR. A major EGFR RNA species of 10-kb (the 5.6-kb band was very faint or absent) was identified in Northern blots using androgen-independent DU145 and PC3 cell lines (Figure 3), as has been described by others (Morris and Dodd, 1990; Ching et al, 1993). EGFR mRNA was undetectable in LNCaPs by Northern analysis due to the low abundance of the message; however, the EGFR mRNA was readily detected by multiplex RT-PCR amplification in these cells. Maximum expression of EGFR mRNA was found at 6–8 h in time course experiments at an optimum ligand concentration of 4 nM EGF and TGF-α. Preliminary experiments also determined that the increase in EGFR mRNA expression induced by 4 nM EGF and TGF-α was similar using cells cultured in either 10% FCS or in serum-free conditions (not shown). Thus, with subsequent experiments, cells were serum starved for 24 h prior to addition of ligand. In LNCaP cells, EGF and TGF-α induced a significant three- to fourfold and two- to threefold up-regulation of EGFR mRNA respectively (Figure 3). EGFR mRNA was significantly up-regulated by six- to eightfold and three- to fourfold in DU145 cells, and two- to threefold and fivefold in PC3 cells with EGF and TGF-α respectively (Figure 3). The ligand-induced increase in EGFR mRNA was mostly back to basal levels by 12 h (data not shown). EGF had greatest effect on EGFR mRNA in LNCaP and DU145 cells whilst TGF-α produced the largest increase in PC3 cells (Figure 3). When all three cells lines were incubated with CHX, EGFR mRNA increased five- to sixfold, suggesting involvement of a labile protein in the regulation of EGFR mRNA turnover (data not shown). These results demonstrated ligand- and cell-specific regulation of EGFR mRNA expression in each of the three prostate cancer cell lines.

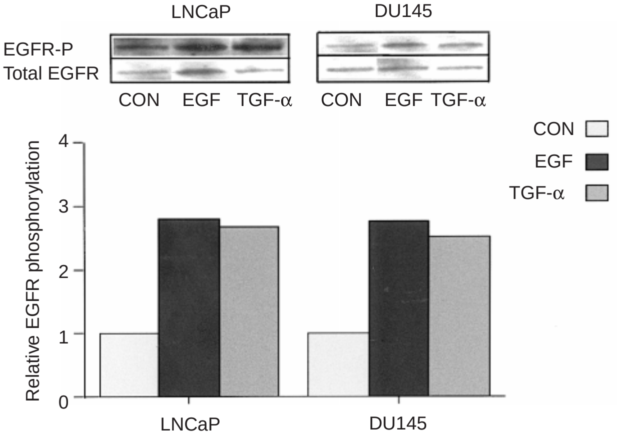

**Figure 2** Phosphorylation of the EGFR by EGF and TGF-α. LNCaP and DU145 cells were serum starved for 24 h, incubated with 4 nM EGF or TGF-α for 15 min, the lysate immunoprecipitated with EGFR antibody (see Materials and Methods), electrophoresed on a SDS-PAGE gel, transferred to nitrocellulose, before immunoblotting with either an antiphosphotyrosine or EGFR antibody and detection of the bands by ECL. EGFR-P, tyrosine phosphorylated EGFR detected using the antiphosphotyrosine antibody. Total EGFR, EGFR protein detected using EGFR antibody. CON, control

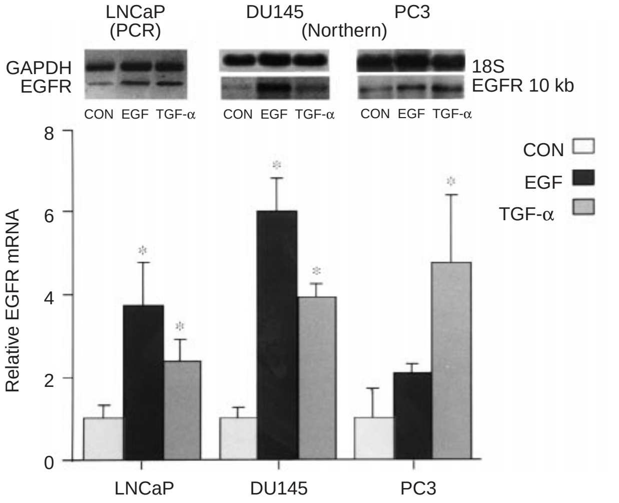

**Figure 3** Effect of EGF and TGF-α on regulation of EGFR mRNA in LNCaP, DU145 and PC3 cells. Total RNA extracted from cells treated for 8 h with 4 nM of either EGF or TGF-α was fractionated on 1% agarose, transferred to nylon membrane and hybridized to a $^{32}$P labelled EGFR-specific cDNA probe for DU145 and PC3 samples. The blot was normalized with a rat 18S cDNA probe. A 10 kb EGFR and 18S message were quantitated by PhosphorImager. For LNCaP cells, 1 μg of total RNA was reverse transcribed with AMV-RT and subjected to multiplex PCR with EGFR and GAPDH primers. The bands were quantitated using ImageQuant software. Each value in the bar chart is shown relative to an arbitrary value of 1 at zero time and is the mean of at least three experiments performed in duplicate. Error bars denote standard error of the mean. The top panel shows bands from a representative experiment. * represents significant difference relative to control

© Cancer Research Campaign 1999

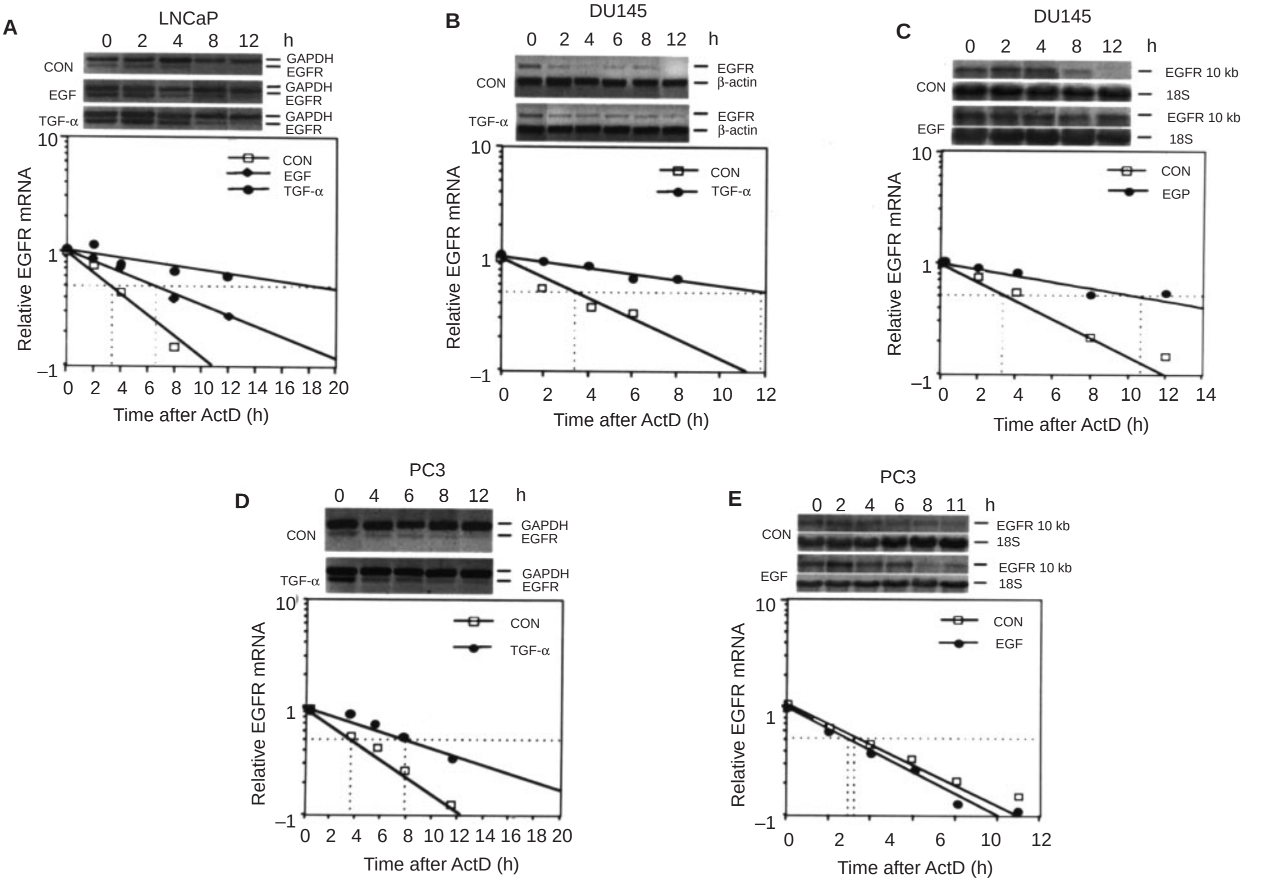

**Figure 4** Effect of EGF and TGF-α on EGFR mRNA stabilization. Cells (LNCaP, DU145, PC3) were grown to 70% confluence, treated with 4 nM EGF or TGF-α for 8 h and chased with 5 μg ml$^{-1}$ actinomycin D for various time intervals. Total RNA was extracted and was subjected to either Northern analysis (**C, E**) or multiplex RT-PCR (**A, B, D**) and quantitated as described in Materials and Methods. The linear regression analysis of EGFR mRNA half life is representative of at least three experiments performed in duplicate. The top panels show bands from individual representative experiments. CON, control. (**A**) LNCaP cells. Multiplex PCR of AMV-reverse transcribed total RNA using EGFR and GAPDH primers. (**B**) DU145 cells and TGF-α. Multiplex PCR of AMV-reverse transcribed total RNA using EGFR and β-actin primers. (**C**) DU145 cells and EGF. Total RNA (12 μg) extracted from DU145 cells was analysed by Northern blotting using $^{32}$P labelled EGFR-specific and rat 18S cDNA probes. (**D**) PC3 cells and TGF-α. Multiplex PCR of AMV-reverse transcribed total RNA using EGFR and GAPDH primers. (**E**) PC3 cells and EGF. Total RNA (12 μg) extracted from PC3 cells was analysed by Northern blotting using $^{32}$P labelled EGFR-specific and rat 18S cDNA probes

### Regulation of EGFR mRNA turnover by EGF and TGF-α in prostate cancer cells

To establish whether the observed EGF- and TGF-α-induced increase in EGFR mRNA expression was due to a change in EGFR mRNA turnover, we used actinomycin D (ActD) pulse chase. Preliminary studies on cell survival in the presence of ActD using trypan blue staining showed that > 90% cells survived 24 h post-treatment (not shown). Cells were incubated ± EGF or TGF-α, ActD added and EGFR mRNA analysed at various time intervals by either Northern (DU145 and PC3 ± EGF) or multiplex RT-PCR analysis (LNCaP ± EGF/TGF-α; DU145 and PC3 ± TGF-α). Either β-actin (DU145) or glyceraldehyde 3-phosphate dehydrogenase (GAPDH) (LNCaP and PC3) primers were used in the multiplex PCR for normalizing. Our analysis showed that TGF-α stabilized EGFR mRNA half-life from ~ 4 h to > 12 h in LNCaP (Figure 4A) and DU145 (Figure 4B) cells, and to ~ 8 h in PC3 cells (Figure 4D). EGF stabilized EGFR mRNA half-life from ~ 4 to ~ 7 h in LNCaP cells (Figure 4A) and from ~ 4 to > 10 h in DU145 cells (Figure 4C). Little change was observed in PC3 cells with EGF on Northern analysis (Figure 4E). TGF-α induced a more pronounced stabilization of EGFR mRNA in all three cell lines. Thus, EGF and TGF-α stabilized EGFR mRNA at least twofold in all three cell lines except in PC3 cells.

### Regulation of EGFR transcription by EGF and TGF-α in prostate cancer cells

In order to determine the transcriptional contribution to the EGF- and TGF-α-induced modulation of the EGFR mRNA, nuclear run-on assays were performed. Figure 5 shows that EGF and TGF-α increased EGFR mRNA transcription ~ four- and~ twofold in both LNCaP and DU145 cells respectively. In contrast, PC3 cells showed a smaller increase in transcription in response to either ligand (Figure 5). These studies indicated that the predominant effect of EGF to increase EGFR mRNA in LNCaP, DU145 and PC3 cells was at the transcriptional level. For TGF-α, however, the major effect was at the post-transcriptional level, inducing substantial increases in EGFR mRNA stability with a smaller relative increase in transcriptional rate.

© Cancer Research Campaign 1999

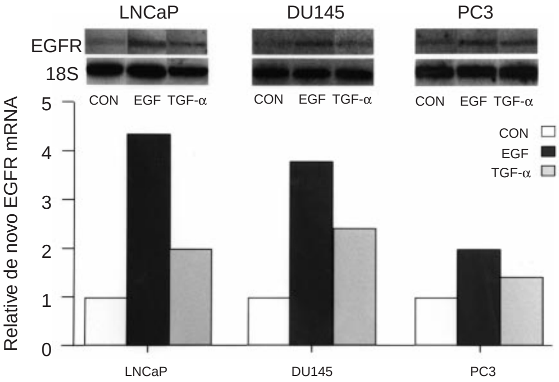

**Figure 5** Transcriptional regulation of EGFR mRNA in LNCaP, DU145 and PC3 cells. Nuclei isolated from cells incubated ± EGF or TGF-α for 8 h were labelled with $^{32}$P-UTP. Equal amounts of radiolabelled RNA were hybridized with EGFR and 18S cDNAs which had been immobilized on nylon membrane. Bands were quantitated, and the ratio of EGFR to 18S transcription calculated (bottom graph). Each value in the graph is relative to 1 for control and is representative of at least two experiments performed in duplicate. The top panel shows the results of an individual experiment. CON, control

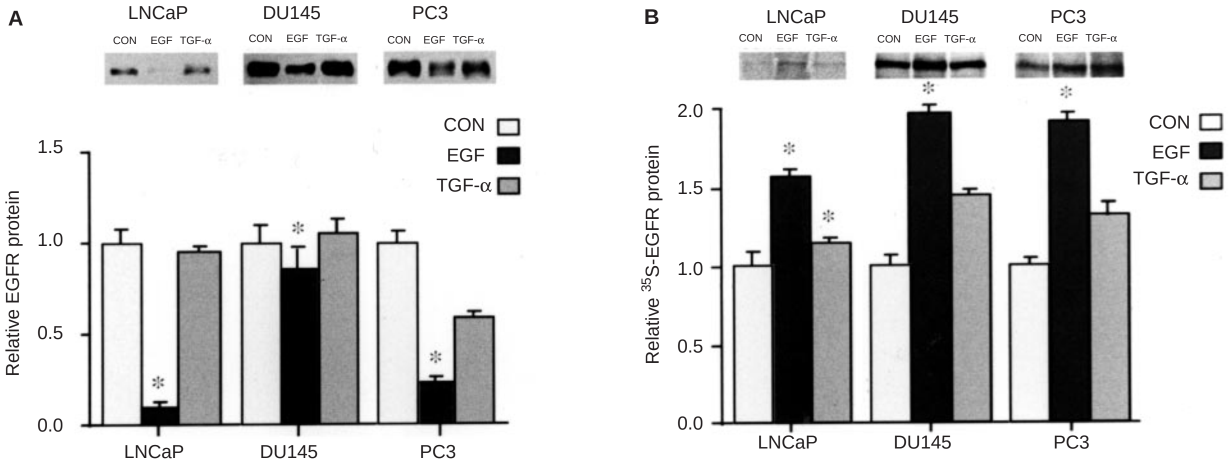

**Figure 6** EGF and TGF-α differentially regulate EGFR protein in LNCaP, DU145 and PC3 cells. (**A**) Total protein assessed by immunoblotting. Total protein was extracted from cells ± EGF or TGF-α, and 20 μg protein/lane was electrophoresed on 7% SDS-PAGE gels, immunoblotted with a sheep anti-human EGFR polyclonal antibody, and detected using enhanced chemiluminescence (ECL). Values shown are relative to an untreated sample of 1 (control) and are representative of at least three experiments performed in duplicate. Error bars denote standard error of the mean. The top panel is representative of an individual experiment. * represents significant difference relative to control. (**B**) De novo protein synthesis by immunoprecipitation. LNCaP, DU145 and PC3 cells were metabolically labelled with L$^{35}$S-methionine/cysteine and incubated ± EGF or TGF-α for 8 h. Samples were immunoprecipitated using a sheep polyclonal anti-human EGFR antibody and Protein A beads. Samples were electrophoresed on a 7% SDS-PAGE gel, dried and quantitated by PhosphorImager. Values shown are relative to an untreated sample of 1 (control) and are representative of at least three experiments performed in duplicate. Error bars denote standard error of the mean. The top panel is representative of an individual experiment. * represents significant difference relative to control

### EGF and TGF-α down-regulate total EGFR protein in a ligand-specific manner

To examine whether the increase in EGFR mRNA was associated with an increase in receptor protein, cells were incubated ± EGF or TGF-α for 6–8 h and EGFR protein analysed by immunoblotting and ECL. The total amount of EGFR protein was at least fivefold greater in untreated samples from DU145 and PC3 cells compared to LNCaP cells (Figure 6A, compare control lanes in LNCaP and DU145). EGF induced a significant decrease in total EGFR protein in LNCaP and PC3 cells (to 14%, and 32% of control respectively), and to a lesser extent in DU145 cells (62%) (Figure 6A). In marked contrast, EGFR protein levels were little changed by TGF-α in LNCaP and DU145 cells (89% and ~ 110% of control, respectively), but showed a significant decrease in PC3 cells (68% of control) (Figure 6A). Thus, major differences exist in the regulation of EGFR protein in these cells, with EGF significantly reducing total EGFR protein compared to TGF-α. Further, it is evident that the EGF- and TGF-α-induced increase in EGFR mRNA described above is not associated with a significant increase in total EGFR protein.

### EGF and TGF-α increase de novo EGFR protein synthesis in prostate cancer cell lines

Our data comparing the regulation of total EGFR protein levels in these prostate cancer cell lines suggested that each ligand was exerting major, yet distinct, effects on EGFR expression at the post-transcriptional level. We next investigated whether the up-regulation of EGFR mRNA was associated with an increase in de novo EGFR protein synthesis. Cells treated with EGF or TGF-α for 7 h were incubated with L$^{35}$S-methionine/cysteine for 1 h and newly synthesized EGFR protein was immunoprecipitated using a

 © Cancer Research Campaign 1999

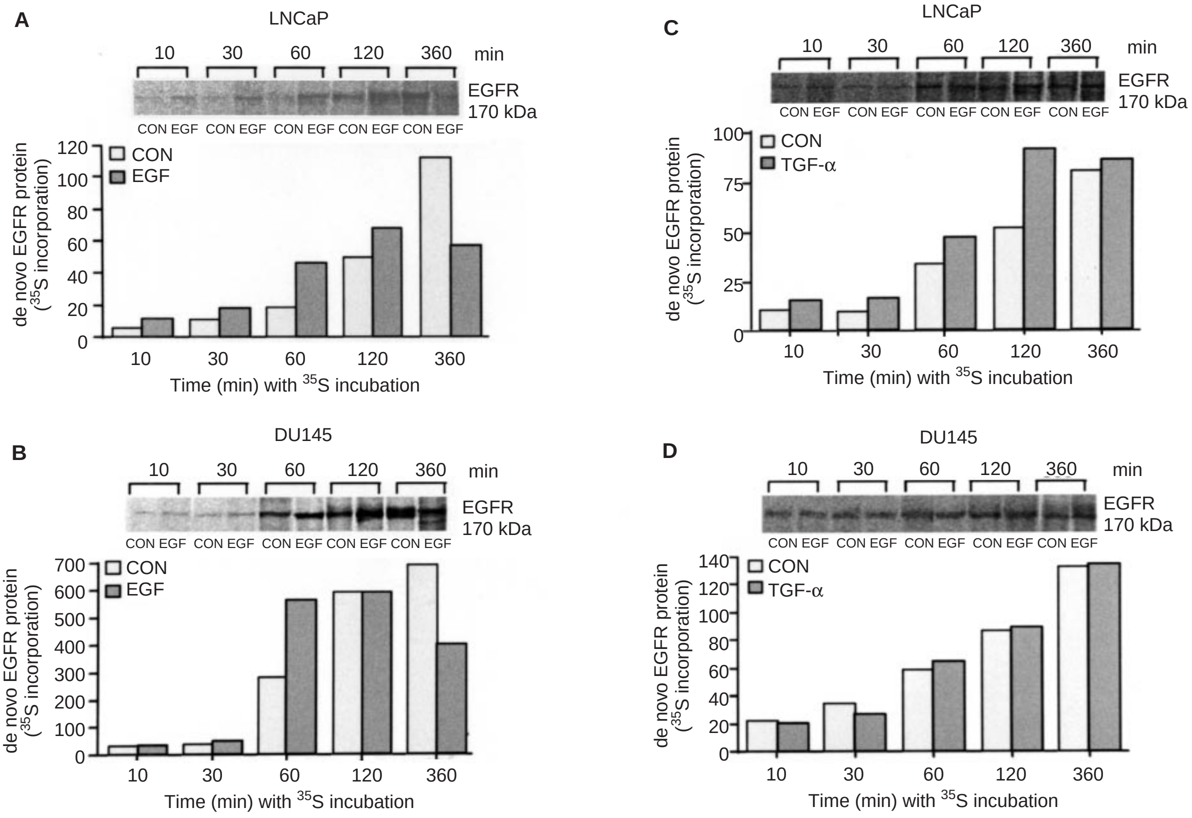

**Figure 7** EGF and TGF-α differentially regulate EGFR protein synthesis in LNCaP and DU145 cells. LNCaP (**A**) and DU145 (**B**) cells were incubated ± EGF (4 nM) or TGF-α (4 nM), metabolically labelled with L$^{35}$S-methionine/cysteine for different time intervals (10, 30, 60, 120 min, 6 h), immunoprecipitated as described above and analysed using a PhosphorImager. Values shown represent relative $^{35}$S incorporation and are representative of two experiments performed in duplicate. The top panels are representative of an individual experiment sheep anti-human EGFR polyclonal antibody as described in Materials and Methods. Figure 6B shows that in LNCaP, DU145 and PC3 cells newly translated EGFR protein levels significantly increased to 1.5-, two- and ~ twofold with EGF, but only 1.2-, 1.5- and 1.3-fold with TGF-α respectively. These data confirmed that the increased EGFR mRNA was associated with an increased synthesis of new EGFR protein. However, although each ligand increased new EGFR protein synthesis, the increase with EGF was significantly greater than with TGF-α. The combination of an EGF-induced increase in new protein synthesis associated with markedly reduced total protein levels suggested that EGF was exerting an additional major effect at the translational and/or post-translational level.

### EGF and TGF-α differentially regulate EGFR protein turnover in prostate cancer cells

To further define the regulation of EGFR protein turnover, we performed time course studies of de novo EGFR synthesis. LNCaP and DU145 cells were incubated with L$^{35}$S-methionine/cysteine for various time intervals (10 min to 6 h) before immunoprecipitation with EGFR antibody, and analysed by SDS-PAGE. Figure 7A and B shows that within 10 min of the addition of EGF a change in rate of EGFR protein synthesis was detectable, which reached a maximum of twofold increase above control by 60 min in both LNCaP (Figure 7A) and DU145 (Figure 7B) cells. However, by 2 h the level of synthesis in control LNCaP and DU145 cells was equal to that in cells treated with EGF. Further, by 6 h the rate of synthesis of EGFR in control cells was at least twofold greater than EGF-treated cells in both LNCaP and DU145 cells. Thus, in contrast to the effect of EGF, in control cells EGFR protein synthesis increased gradually over the 6-h incubation. These data suggest that although EGF increases the de novo protein synthesis rate within the first hour of treatment, the increase is not sustained. This may in part reflect that with the longer times of incubation, we were measuring both synthetic rate and also a component of EGFR decay. We next examined the effect of TGF-α on EGFR protein synthesis in LNCaP and DU145 cells. Figure 7C and D shows that TGFα increased EGFR protein synthesis ~ twofold in LNCaP cells at 120 min (Figure 7C) but little difference was recorded in DU145 cells over control (Figure 7D). Moreover, the EGFR protein levels were sustained and did not decrease below control at 6 h as observed with EGF.

A marked disparity still remained between the EGFR synthetic rate and the total protein data in Figure 6A. To address the possibility that EGF and TGF-α differentially modulated EGFR protein stability, L$^{35}$S-methionine/cysteine pulse chase studies were performed. LNCaP and DU145 cells ± EGF or TGF-α were incubated with $^{35}$S-methionine for 60 min, after which the cells were replenished with regular medium. EGFR protein was immunoprecipitated after various time intervals (0, 30, 60, 120 min, 5 h) and resolved by SDS-PAGE as above. Results in Figure 8A and B

© Cancer Research Campaign 1999 

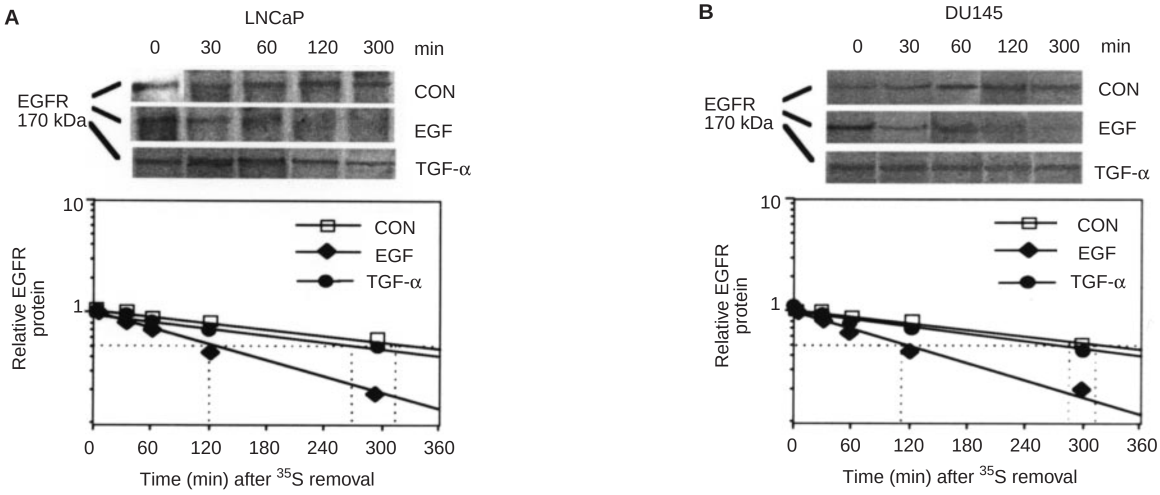

**Figure 8** EGF and TGF-α differentially regulate EGFR protein stability in LNCaP and DU145. LNCaP and DU145 cells were incubated ± EGF (4 nM, **A** and **B** respectively) or TGFα (4 nM, **C** and **D** respectively), metabolically labelled for 1 h with $L^{35}S$-methionine/cysteine, replaced with media without radiolabel and proteins extracted after various time intervals with 0 min as the first time point at media replacement (0, 30, 60, 120, 300 min). Proteins were immunoprecipitated as described above and analysed using a PhosphorImager. Values shown represent relative $^{35}S$ incorporation are representative of experiments performed in duplicate. The top panel represents results from an individual experiment

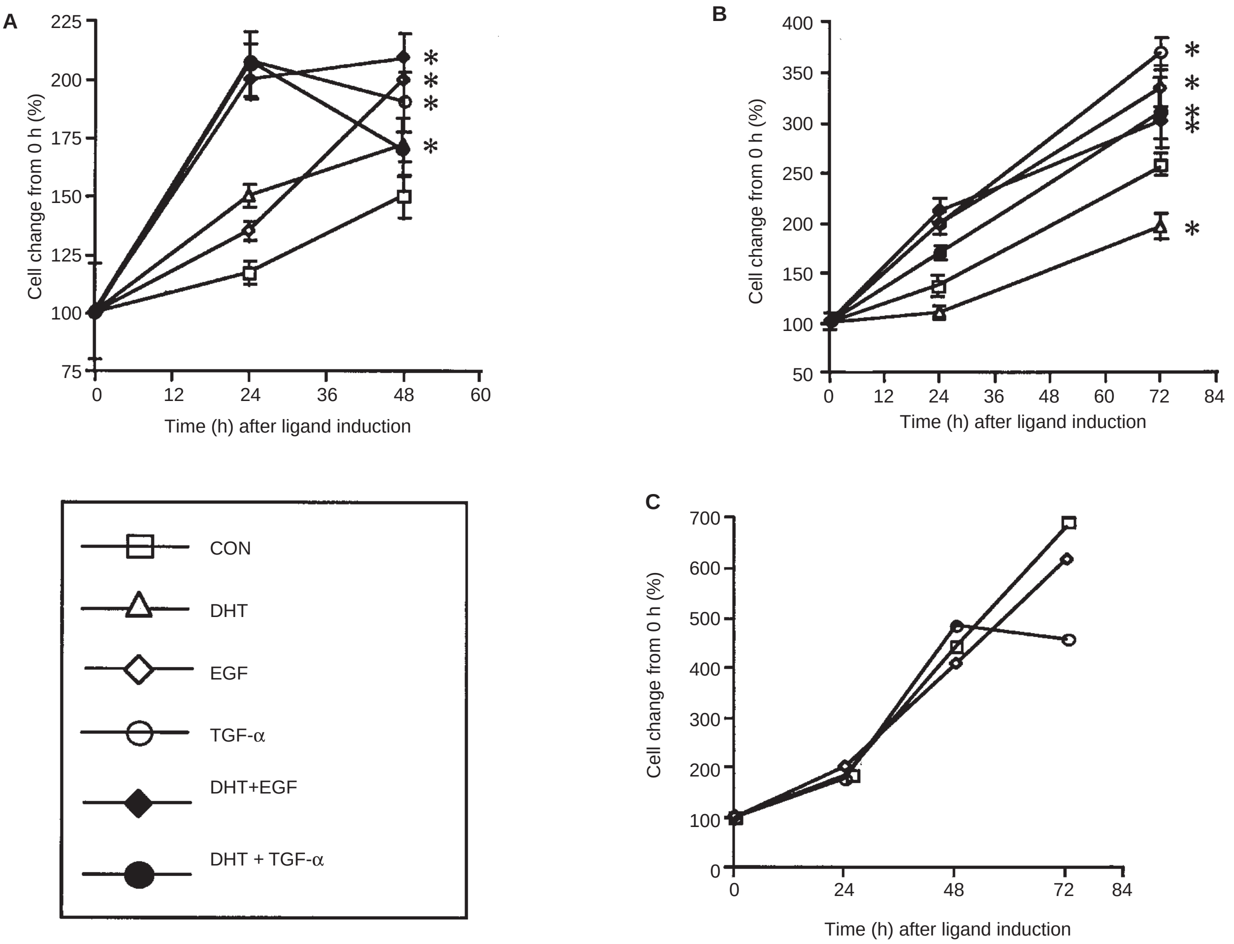

**Figure 9** Regulation of LNCaP, DU145 and PC3 cell proliferation by EGF, TGF-α and DHT. Cells [(LNCaP (**A**), DU145 (**B**) and PC3 (**C**)] were serum starved and treated with 4 nM EGF or TGF-α and/or 10 nM DHT. Cells were trypsinized at various time intervals up to 72 h and counted. The graph represents an average of two experiments performed in triplicate, expressed as percent cell number change from 0 h (time of ligand/hormone addition). Error bars represent standard deviation. * represents significant difference relative to control

 *© Cancer Research Campaign 1999*

shows that EGF induced rapid disappearance of EGFR protein in LNCaP and DU145 cells with a half-life of ~ 120 min, compared to ~ 5 h in control. This rapid decrease in EGFR protein stability within 120 min after treatment with EGF would explain, in part, the low levels of total EGFR protein observed in immunoblotting (Figure 6A). Interestingly, when LNCaP and DU145 cells were treated with TGF-α the rate of disappearance of EGFR protein did not change in either cell line (see Figure 8A, B). The half-life of the EGFR protein remained ~ 5 h in the presence and absence of TGF-α. These results emphasize the complexity of the post-transcriptional control of EGFR expression induced by each ligand.

### Ligand-induced cell proliferation of prostate cancer cells

To monitor the ligand-induced effect on cell proliferation, LNCaP, DU145 and PC3 cells were serum starved for 24 h, treated with EGF or TGF-α and/or DHT for different time intervals, trypsinized and counted. At 24 and 48 h both EGF and TGF-α increased cell numbers in androgen-dependent LNCaP cells significantly above control (~ 1.5–2-fold), and also above the increase induced by DHT (~ 1.2-fold above control) at 48 h (Figure 9A). The combination of DHT and EGF was additive resulting in significant proliferation (~ 1.8-fold) above control. However, the response to DHT and TGF-α together was no greater than with TGF-α alone. In the androgen-independent DU145 cell line, EGF and TGF-α both significantly increased cell proliferation one- to twofold above control levels at 24 and 72 h (Figure 9B). DHT alone or in combination with EGF or TGF-α had no positive effect on cell proliferation (Figure 9B). In PC3 cells neither ligand induced growth above control at 24 or 48 h (Figure 9C). These results indicate that EGF and TGF-α induce significant cell proliferation in both androgen-dependent (LNCaP) and androgen-independent (DU145) cells. Furthermore, it illustrates the potential for the EGF/TGF-α–EGFR pathway to contribute to prostate cancer cell at both relatively early (androgen-responsive) and more advanced (androgen-unresponsive) stage disease.

### DISCUSSION

To investigate the molecular mechanisms involved in the modulation of EGFR-mediated growth in prostate cancer, we examined the effect of EGF and TGF-α on the regulation of EGFR expression in androgen-dependent (LNCaP) and -independent (DU145 and PC3) prostate cancer cell lines. Our results demonstrate that EGF and TGF-α induce distinct mechanisms to up-regulate EGFR expression, which are both ligand- and cell-specific (see Table 1). With the exception of PC3 cells, each ligand differentially up-regulated both EGFR mRNA stability and transcription. In LNCaP and DU145 cells, EGF stabilized EGFR mRNA ~ twofold, whereas no significant effect was observed in PC3 cells. TGF-α, however, stabilized EGFR mRNA ~ twofold greater than EGF in all three cell lines. The converse was found at the transcriptional level, where EGF increased transcription in LNCaP and DU145 cells ~ twofold greater than TGF-α. Thus, the predominant effect of TGF-α at the mRNA level is post-transcriptional in all three cell lines, whereas for EGF the major contribution is at the transcriptional level (Table 1). The CHX-induced increase in EGFR mRNA indicated that a labile protein may be involved in the maintenance of EGFR mRNA turnover. Furthermore, it raised the possibility that regulation of EGFR mRNA decay may be closely coupled to its translation. Several examples have been previously described linking mRNA decay to translation (Peltz and Jacobson, 1992; Sachs, 1993; Decker and Parker, 1994).

mRNA decay is now recognized as a major control point in the regulation of gene expression. Our results represent the first to demonstrate the importance of changes in EGFR mRNA turnover in the prostate. Previous reports in other tissues have described changes in EGFR mRNA stability in response to various ligands. The EGF-induced increase in EGFR mRNA in breast MDA-468 (Fernandez-Pol et al, 1989) and epidermoid KB (Jinno et al, 1988) cancer cells is, in part, due to increased stability of EGFR mRNA. In addition, thyroid hormone dramatically reduces EGFR mRNA stability in A431 cells (Kesavan et al, 1991). The mechanisms involved in facilitating increased stability of the EGFR mRNA in prostate cancer cells are unknown but may involve RNA–protein interactions between *cis*-acting mRNA stability modifying regions and *trans*-acting EGFR RNA binding proteins. Many short-lived mRNAs, including several of the cytokines and proto-oncogenes, contain an AU-rich region (AU-R, typically AUUUA repeats) in the 3′-UTR which can confer metabolic instability by targeting these mRNAs for rapid degradation (Greenberg and Belasco 1993). Interestingly, the 3′-UTR of EGFR mRNA contains two AU-rich regions and three AUUUA pentamers (see Figure 1). One or more of these motifs may represent a target for growth factor-regulated AU-rich RNA binding factors (AUBFs). This EGFR mRNA–protein complex may function to protect the mRNA against degradation, thereby increasing the mRNA stability in the presence of ligand. A more stable EGFR mRNA would promote increased EGFR protein production and cellular proliferation, and favour the development of an autocrine loop. Current studies are underway to define the *cis–trans* EGFR RNA–protein interactions

**Table 1** Differential regulation of EGFR expression by EGF and TGF-α in LNCaP, DU145 and PC3 human prostate cancer cells

| | Total mRNA | Total protein | De novo protein | EGFR mRNA stability | EGFR transcription | Cell proliferation | |
|---|---|---|---|---|---|---|---|
| LNCaP | 4 | 0.14 | 1.5 | >2 | 4 | 1.5 | EGF |
| DU145 | 6 | 0.62 | 2 | 2 | 4 | 1.5 | EGF |
| PC3 | 2 | 0.32 | 2 | 1 | 2 | 1 | EGF |
| LNCaP | 2 | 0.89 | 1.2 | 3–4 | 2 | 2 | TGF-α |
| DU145 | 2 | 1.1 | 1.5 | 3–4 | 2 | 2 | TGF-α |
| PC3 | 5 | 0.68 | 1.3 | 1.2 | 1.5 | 1 | TGF-α |

Regulation of EGFR expression at transcriptional, post-transcriptional and translational levels by EGF and TGF-α in androgen-dependent (LNCaP) and -independent (DU145, PC3) prostate cancer cell lines. Values are relative to a control level of 1 prior to ligand addition.

© Cancer Research Campaign 1999

in human prostate cancer cells that control EGFR mRNA stability.

The mechanisms by which EGF and TGF-α produced low levels of total EGFR protein, in the context of elevated levels of EGFR mRNA in LNCaP, DU145 and PC3 cells proved to be of considerable interest. The down-regulation in total protein with EGF was most pronounced in LNCaP and PC3 cells. In marked contrast, there was only a slight change in total EGFR protein levels in LNCaP and DU145 cells with TGF-α. The increase in EGFR mRNA levels and stability was associated with increased synthesis of EGFR protein (see Table 1). However, pulse chase labelling studies indicated that the down-regulation of EGFR protein induced by EGF was the result of rapid disappearance of the receptor protein, which was not compensated for by the increased level of EGFR mRNA, its stabilization or enhanced translation of the protein. Thus, EGF preferentially induced rapid EGFR protein decay in these human prostate cancer cell lines.

Increased EGFR mRNA expression with decreased protein levels has been previously reported in various cell types in the presence of exogenous EGF. For example, in the WB cell line from rat hepatic epithelium, a three- to fivefold EGF-induced increase in EGFR mRNA was associated with a down-regulation in EGFR protein (Earp et al, 1986). EGF has also been reported to down-regulate EGFR protein in human epidermoid A431 and KB cells (Kawamoto et al, 1983; Lifshitz et al, 1983; Clark et al, 1985) and breast cancer cells (Kudlow et al, 1986; Bilous et al, 1992). In prostate tumours high levels of EGFR mRNA have been reported to vary inversely with EGFR protein levels (Turkeri et al, 1994). Further, although EGFR mRNA has been detected at higher levels in prostate cancer specimens (Ching et al, 1993), exogenous EGF has been shown to down-regulate EGFR protein expression (Maddy et al, 1989; Turkeri et al, 1994). The work presented here adds support to these observations, defines the molecular mechanisms involved and illustrates the differences between the action of EGF and TGF-α at the post-transcriptional level.

Recent studies have provided insight into the pathways involved in the intracellular trafficking of the EGFR, and similar molecular mechanisms may be operative in prostate cancer cells. Recent reports suggest that alternative intracellular routing of the EGFR and its ligands may contribute to a broad range of signal transduction, and consequently have a profound impact on cellular proliferation. Previous studies demonstrated that EGF induced internalization of the EGF–EGFR complex to clathrin-coated pits, movement to receptosomes and delivery to lysosomes facilitating complete degradation of EGFR within 120 min (Beguinot et al, 1984, 1985). Subsequent studies indicated that ligand occupancy of the receptor was critical for efficient targetting. Wiley at al (1991) correlated the EGF-induced EGFR down-regulation to occupancy-induced endocytosis. This was due to an increase in receptor targetting to lysosomes and subsequent degradation by increasing the pool of receptors at steady state. Interestingly, ligand-occupied kinase-active EGFRs were internalized through a high affinity endocytic system at rates up to ten times faster than empty receptors, suggesting a central role for ligand in the signalling. Several studies have since examined differences between the effects of EGF and TGF-α on receptor degradation. EGF was shown to be resistant to dissociation from the EGFR in endosomes (French et al, 1995), whilst TGF-α rapidly dissociated from the EGFRs (Ebner and Derynck, 1991) resulting in more efficient targetting of EGFRs to lysosomes with EGF than with TGF-α. This would lead to enhanced biological activity due to repeated presentation of recycled EGFRs at the cell surface, resulting in multiple rounds of signalling. Most recently, Waterman et al (1998) demonstrated that EGF and TGF-α had markedly different effects on receptor down-regulation in CHO cells. They showed that EGF-stimulated EGFRs were destined for rapid lysosomal degradation. In contrast, EGFRs bound by TGF-α underwent rapid endocytosis but were shunted down a different pathway leading to receptor recycling with only limited down-regulation. Consistent with this, the mitogenic activity of TGF-α was superior to that of EGF in this system, reinforcing the concept that differential intracellular EGFR routing may play an important role in the regulation of mitogenic signals.

Recent studies using a HeLa cell line defective in clathrin-coated vesicles, illustrated the importance of this pathway for intracellular EGFR trafficking (Vieira et al, 1996). In wild-type HeLa cells, EGFR was endocytosed and degraded with a half-life of 30 min. However, in these mutant HeLa cells, approximately 80% of the EGFRs were detected even after 2 h. The recent discovery of a sorting protein, nexin-1 (SNX1), which sorts EGFR to lysosomes (Kurten et al, 1996), provided clues to the potential molecular mechanism(s) involved. Overexpression of SNX1 reduced EGFR expression at the cell surface in CV1 cells and increased EGF-induced EGFR degradation. Whether SNX1 is differentially regulated by EGF and TGF-α, and its role in prostate cancer cells, is yet to be determined. However, our data are consistent with the concept that EGF and TGF-α differentially regulate intracellular EGFR trafficking. We propose that, in prostate cancer cells, EGF preferentially diverts the internalized EGF–EGFR complex to endosomes and lysosomes, whilst TGF-α dissociates from the complex and recycles EGFR back to the cell membrane (see Figure 10). Further work is in progress to investigate this thesis.

The variety of erbB receptors (EGFR, erbB2, erbB3 and erbB4) provides another level of complexity that adds to the diversity of the erbB signalling network (Burden and Yarden, 1997). Each ligand molecule is bivalent with a high affinity site for binding the ‘primary receptor’, and a low affinity site with broad specificity that facilitates recruitment of other members of the erbB receptor family into heterodimers, resulting in differential and effective signalling (Tzahar et al, 1997). How this system might impact on the observations that we found in the present study is yet to be determined. However, based on the heterodimer capacity of the erbB family, and the recent work of Waterman et al (1998) in which Neu differentiation factor (NDF) has a similar effect to TGF-α to drive erbB3 receptor recycling and result in a more potent mitogenic response, elucidation of the mechanisms and role of heterodimer partners in the intracellular signalling of human prostate cancer cells may well provide new insight into mitogenesis and cellular proliferation.

Although several groups have characterized a TGF-α–EGFR autocrine loop in prostate cancer cells (Wilding et al, 1989; Connolly and Rose, 1990; Liu et al, 1993; Xie et al, 1995), the molecular mechanisms governing the development and maintenance of the loop have not been determined. In both LNCaP and DU145 cells, EGF and TGF-α are synthesized and secreted into the medium. The regulation of EGF synthesis by androgens may be altered in human prostate cancer. The weight of evidence suggests that in LNCaP cells there is a change to TGF-α and EGFR production, resulting in continuous autocrine stimulation of

© Cancer Research Campaign 1999

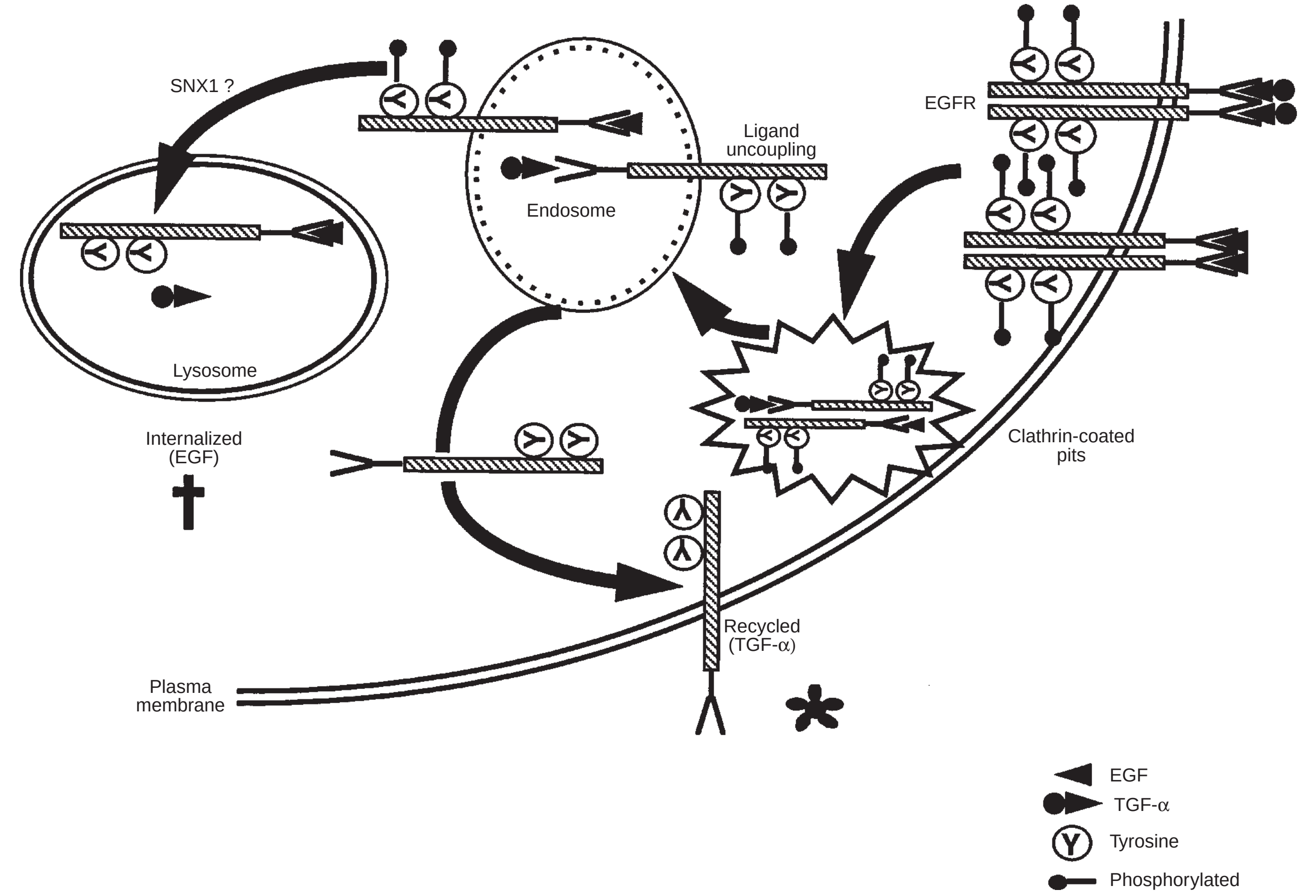

**Figure 10** Proposed model for differential sorting and transport of EGFR by TGF-α and EGF in prostate cancer cells. The active ligand–receptor complex (TGF-α–EGFR or EGF–EGFR) is internalized to clathrin coated pits and then to endosomes. For TGF-α, the receptor–ligand complex undergoes dissociation of the ligand and the inactive EGFR is recycled back to plasma membrane. For EGF, the ligand–receptor complex undergoes transcytosis and lysosomal degradation. Dephosphorylation and inactivation of the EGFR tyrosine kinase occurs in the endosomal compartments. The sorting protein nexin (SNX1) may play a role to facilitate targetting of the EGF–EGFR complex to lysosomes

LNCaP cell proliferation. Importantly, this autocrine loop persists in androgen-insensitive DU145 cells, providing a case for support of prostate cancer cell growth in part, by TGF-α, following androgen withdrawal. Our data demonstrate that EGF and TGF-α preferentially regulate expression at different steps of the EGFR synthetic pathway. The net result of these effects is a greater increase of EGFR protein concentration with TGF-α compared to EGF. This would favour establishment of an autocrine loop in these cells. Previous work has suggested that prostate cancer cells undergo a 'switch' in secretion from EGF to TGF-α which is associated with a poor prognosis (Ching et al, 1993; Steiner, 1993) and more advanced disease. Our data are consistent with these clinicopathologic findings and they provide a molecular explanation why autocrine secretion of TGF-α, rather than EGF, would lead to sustained EGFR expression and increased prostate cancer cell proliferation and growth.

EGF and TGF-α induced cell proliferation in LNCaP and DU145 cells, but not in PC3 cells. Interestingly, the increase in cell proliferation was significantly greater in LNCaP cells with EGF and TGF-α than with DHT and there was no additive effect of TGF-α when combined with DHT. These data are consistent with others who have reported a ligand-induced increase in cell proliferation in DU145 (Connolly and Rose, 1990, 1991) and in LNCaP of ~ twofold with EGF and TGF-α, and ~ threefold with the synthetic androgen, R1881 (Schuurmans et al, 1991). However, our results differ from reports where TGF-α displayed little response in LNCaP cells, yet increased PC3 cell growth by 35% (Hofer et al, 1991; Carruba et al, 1994). The differences that we observed in proliferative responses to EGF and TGF-α between the two EGFR overexpressing androgen-unresponsive cell lines (DU145 and PC3) are also of note. For PC3 cells these data suggest that growth proliferation (~ fourfold over 48 h) is independent of EGF, TGF-α and androgens, and implicates involvement of other growth factors, such as insulin-like growth factor (IGF) (Steiner, 1993; Byrne et al, 1996), TGFβ and fibroblast growth factor (FGF) (Steiner, 1993; Steiner and Barrack, 1992). However, PC3 cell proliferation is inhibited by antibodies to TGF-α and EGFR, suggesting that the TGF-α–EGFR pathway may be permissive for growth in these cells (Hofer et al, 1991). In keeping with these observations, one group has shown that the major effect of EGF in PC3 cells is to promote invasion, rather than to stimulate cell proliferation (Jarrard et al, 1994).

LNCaP cells express far fewer EGFRs than the two androgen-unresponsive DU145 and PC3 cell lines (Wilding et al, 1989).

© Cancer Research Campaign 1999 

However, the regulation of EGFR expression and proliferative responses to EGF and TGF-α that we observed in LNCaP and DU145 cells were similar. Thus, in androgen-responsive prostate cancer cells, the EGFR pathway may make a significant contribution to cellular proliferation. As a consequence, blocking the EGFR pathway earlier in the treatment of prostate cancer when tumours are androgen-sensitive may provide a therapeutic advantage. Studies to address the role of dual anti-androgen and anti-EGFR treatment strategies to reduce prostate cancer cell growth will be required to address this issue.

In summary, these data demonstrate the complexity of the regulation of EGFR expression by TGF-α and EGF in prostate cancer cells. EGFR transcription, mRNA decay, protein synthesis and protein decay are regulated differentially by EGF and TGF-α and in a cell-specific manner. These studies provide novel insight into differences of action of each ligand at the molecular level. We have defined the molecular mechanisms which indicate a preferred role for TGF-α in the maintenance of an autocrine loop for proliferative growth. In this context, a change in autocrine synthesis from EGF to TGF-α during progression from hormone-responsive to advanced prostate carcinomas would result in a net increase in EGFR protein expression and could confer a significant growth advantage to the cells. Furthermore, these data emphasize the need to consider blocking the EGFR proliferative pathway earlier in the androgen-responsive phase of human prostate cancer in order to improve therapeutic outcomes.

### ACKNOWLEDGEMENTS

The authors are grateful to Roger Davis for providing the pbluescript-EGFR plasmid, to Dr Peta Tilbrook for providing phosphotyrosine antibodies and to the Medical Research Fund of Western Australia (MEDWA) for supporting this work.

### REFERENCES

Ausubel FM, Brent R, Kingston RE, Moore DD, Seidman JG, Smith JA and Struhl K (eds) (1994) Identification of newly transcribed RNA. In: *Current Protocols in Molecular Biology*. Wiley: Toronto

Beguinot L, Lyall RM, Willingham MC and Pastan I (1984) Down-regulation of the epidermal growth factor receptor in KB cells is due to receptor internalisation and subsequent degradation in lysosomes. *Proc Natl Acad Sci USA* **81**: 2384–2388

Beguinot L, Hanover JA, Ito S, Richert ND, Willingham MC and Pastan I (1985) Phorbal esters induce transient internalisation without degradation of unoccupied epidermal growth factor receptors. *Proc Natl Acad Sci USA* **82**: 2774–2778

Bilous M, Milliken J and Mathijs JM (1992) Immunocytochemistry and in situ hybridization of epidermal growth factor receptor and relation to prognostic factors in breast cancer. *Eur J Cancer* **28**: 1033–1037

Brass AL, Barnard J, Patai BL, Salvi D and Rukstalis DB (1995) Androgen up-regulates growth factor receptor expression and binding affinity in PC3 cell lines expressing the human androgen receptor. *Cancer Res* **55**: 3197–3203

Burden S and Yarden Y (1997) Neuregulins and their receptors: a versatile signaling module in organogenesis and oncogenesis. *Neuron* **18**: 847–855

Byrne RL, Leung H and Neal DE (1996) Peptide growth factors in the prostate as mediators of stromal epithelial interaction. *Br J Urol* **77**: 627–633

Carruba G, Leake RE, Rinaldi F, Chalmers D, Comito L, Sorci C, Pavone-Macaluso M and Castagnetta LAM (1994) Steroid-growth factor interaction in human prostate cancer. Part 1: Short-term effects of transforming growth factors on growth of human prostate cancer cells. *Steroids* **59**: 412–420

Ching KZ, Ramsey E, Pettigrew N, D'Cuhna R, Jason M and Dodd JG (1993) Expression of mRNA for epidermal growth factor, transforming growth factor-alpha and their receptor in human prostate tissue and cell lines. *Mol Cell Biochem* **126**: 151–158

Clark AJ, Ishii S, Richert N, Merlino GT and Pastan I (1985) Epidermal growth factor stimulates the expression of its own receptor. *Proc Natl Acad Sci USA* **82**: 8374–8378

Connolly JM and Rose DP (1990) Production of epidermal growth factor and transforming growth factor-α by androgen-responsive LNCaP human prostate cancer cell line. *Prostate* **16**: 209–218

Connolly JM and Rose DP (1991) Autocrine regulation of DU145 human prostate cancer cell growth by epidermal growth factor-related polypeptides. *Prostate* **19**: 173–180

Davies P and Eaton C (1989) Binding of epidermal growth factor by human normal, hypertrophic, and carcinomatous prostate. *Prostate* **14**: 123–132

Decker CJ and Parker R (1994) Mechanisms of mRNA degradation in eukaryotes. *Trends Biochem Sci* **19**: 336–340

Earp HS, Austin KS, Blaisdall J, Rubin RA, Nelson KG, Lee LW and Grisham JW (1986) Epidermal growth factor stimulates EGF receptor synthesis. *J Biol Chem* **261**: 4777–4780

Ebner R and Derynck R (1991) Epidermal growth factor and transforming growth factor-alpha: differential intracellular routing and processing of ligand-receptor complexes. *Cell Regul* **2**: 599–612

Ennis BW, Valverius EM, Bates SE, Lippman ME, Bellot F, Kris R, Schlessinger J, Masui H, Goldenberg A, Mendelsohn J and Dickson RB (1989) Anti-epidermal growth factor receptor antibodies inhibit the autocrine-stimulated growth of MDA468 human breast cancer cells. *Mol Endocrinol* **3**: 1830–1838

Fernandez-Pol JA, Hamilton PD and Klos DJ (1989) Transcriptional regulation of proto-oncogene expression by epidermal growth factor, transforming growth factor β1, and triiodothyronine in MDA468 cells. *J Biol Chem* **264**: 4151–4156

French AR, Tadaki DK, Niyogi SK and Lauffenburger DA (1995) Intracellular trafficking of epidermal growth factor family ligands is directly influenced by the pH sensitivity of the receptor/ligand interaction. *J Biol Chem* **270**: 4334–4340

Glynne-Jones E, Goddard L and Harper ME (1996) Comparative analysis of mRNA and protein expression for epidermal growth factor receptor and ligands relative to the proliferative index in human prostate tissue. *Hum Pathol* **27**: 688–694

Greenberg ME and Belasco JG (1993) Control of degradation of labile protooncogene and lymphokine mRNAs. In: *Control of mRNA Stability*, Belasco JG and Brawerman G (eds), pp. 199–218. Academic Press: London

Gullick WJ (1991) Prevalence of aberrant expression of the epidermal growth factor receptor in human cancers. *Br Med Bull* **47**: 87–96

Hanover JA, Beguinot L, Willingham MC and Pastan IH (1985) Transit of receptors for epidermal growth factor and transferrin through clathrincoated pits. Analysis of the kinetics of receptor entry. *J Biol Chem* **260**: 15938–15945

Hofer DR, Sherwood ER, Bromberg WD, Mendelsohn J and Lee C (1991) Autonomous growth of androgen-independent human prostatic carcinoma cells: role of transforming growth factor α. *Cancer Res* **51**: 2780–2785

Jarrard DF, Blitz BF, Smith RC, Patai BL and Rukstalis DB (1994) Effect of epidermal growth factor on prostate cancer cell line PC3 growth and invasion. *Prostate* **24**: 46–53

Jinno Y, Merlino GT and Pastan I (1988) A novel effect of EGF on mRNA stability. *Nucl Acid Res* **16**: 4957–4966

Kawamoto T, Sato JD, Le A, Polikoff J, Sato GH and Mendelsohn J (1983) Growth stimulation of A431 cells by epidermal growth factor: identification of high affinity receptors for epidermal growth factor by an anti-receptor monoclonal antibody. *Proc Natl Acad Sci USA* **80**: 1337–1341

Kesavan P, Mukhopadhyay S, Murphy S, Rengaraju M, Lazar MA and Das M (1991) Thyroid hormone decreases the expression of epidermal growth factor receptor. *J Biol Chem* **266**: 10282–10286

Kudlow JE, Cheung C-YM and Bjorge JD (1986) Epidermal growth factor stimulates its own receptor in a human breast cancer cell line. *J Biol Chem* **261**: 4134–4138

Kurten RC, Cadena DL and Gill GN (1996) Enhanced degradation of EGF receptors by a sorting nexin, SNX1. *Science* **272**: 1008–1010

Lifshitz A, Lazar CS, Buss JE and Gill GN (1983) Analysis of morphology and receptor metabolism in clonal variant A431 cells with differing growth responses to epidermal growth factor. *J Cell Physiol* **115**: 235–242

Lippman ME (1993) The development of biological therapies for breast cancer. *Science* **259**: 631–632

Liu X-H, Wiley HS and Miekle AW (1993) Androgens regulate proliferation of human prostate cancer cells in culture by increasing transforming growth factor-α and epidermal growth factor/TGF-α receptor. *J Clin Endocrinol Metab* **77**: 1472–1478

Maddy SQ, Chisholm GD, Busuttil A and Habib FK (1989) Epidermal growth factor receptors in human prostate cancer: correlation with histological differentiation of the tumor. *Br J Cancer* **60**: 41–44

© Cancer Research Campaign 1999

Mendelsohn J (1992) Epidermal growth factor receptor as a target for therapy with antireceptor monoclonal antibodies. *J Natl Cancer Inst Monogr* **13**: 125–131
Modjtahedi H and Dean C (1994) The receptor for EGF and its ligands: expression, prognostic value and target for therapy in cancer. *Int J Oncol* **4**: 277–296
Morris GL and Dodd JG (1990) Epidermal growth factor receptor mRNA levels in human prostatic tumors and cell lines. *J Urol* **143**: 1272–1274
Nomura AMY and Kolonel N (1991) Prostate cancer: a current perspective. *Am Epidemiol* **18**: 200–226
Peltz SW and Jacobson A (1992) Regulation of mRNA turnover in eukaryotic cells. *Curr Opin Cell Biol* **4**: 979–983
Prewett M, Rockwell P, Rockwell RF, Giorgio NA, Mendelsohn J, Scher HI and Goldstein NI (1997) The biologic effects of C225, a chimeric monoclonal antibody to the EGFR, on human prostate carcinoma. *J Immunother* **19**: 419–427
Sachs AB (1993) Messenger RNA degradation in eukaryotes. *Cell* **74**: 413–421
Schuurmans ALG, Bolt J and Mulder E (1988) Androgens stimulate both growth rate and epidermal growth factor receptor activity of the human prostate tumor cell line LNCaP. *Prostate* **12**: 55–63
Schuurmans ALG, Bolt J, Veldscholte J and Mulder E (1991) Regulation of growth of LNCaP human prostate tumor cells by growth factors and steroid hormones. *J Steroid Biochem Molec Biol* **40**: 193–197
Steiner MS (1993) Role of peptide growth factors in the prostate: a review. *Urology* **42**: 99–110
Steiner MS and Barrack ER (1992) Transforming growth factor β-1 overproduction in prostate cancer: effects of growth in vivo and in vitro. *Mol Endocrinol* **6**: 15–25
Tilbrook PA, Bittorf T, Busfield SJ, Chappell D and Klinken SP (1996) Disrupted signalling in a mutant J2E cell line that shows enhanced viability, but does not proliferate or differentiate, with erythropoietin. *J Biol Chem* **271**: 3453–3459
Todaro GJ, Fryling C and DeLarco JE (1979) Transforming growth factors produced by certain human tumor cells: polypeptides that interact with epidermal growth factor receptors. *Proc Natl Acad Sci USA* **77**: 5258–5262
Traish AM, Wotiz HH (1987) Prostatic epidermal growth factor receptors and their regulation by androgens. *Endocrinology* **121**: 1461–1467
Turkeri LN, Sakr WA, Wykes SM, Grignon DJ, Pontes JE and Macoska JA (1994) Comparative analysis of epidermal growth factor receptor gene expression and protein product in benign, premalignant, and malignant prostate tissue. *Prostate* **25**: 199–205
Tzahar E, Pinkas-Kramarski R, Moyer J, Klapper LN, Alroy I, Levkowitz G, Shelly M, Henis S, Eisenstein M, Ratzkin BJ, Sela M, Andrews GC and Yarden Y (1997) Bivalence of EGF-like ligands drives the ErbB signaling network. *EMBO J* **16**: 4938–4950
Vieira AV, Lamaze C and Schmid SL (1996) Control of EGF receptor signalling by clathrin-mediated endocytosis. *Science* **274**: 2086–2089
Waterman H, Sabanai I, Geiger B and Yarden Y (1998) Alternative intracellular routing of erbB receptors may determine signaling potency. *J Biol Chem* **273**: 13819–13827
Wilding G, Valverius E, Knabbe C and Gelmann EP (1989) Role of transforming growth factor-α in human prostate cancer cell growth. *Prostate* **15**: 1–12
Wiley HS, Herbst JJ, Walsh BJ, Lauffenburger DA, Rosenfeld MG, Gill GN (1991) The role of tyrosine kinase activity in endocytosis, compartmentation, and down-regulation of the epidermal growth factor receptor. *J Biol Chem* **266**: 11083–11094
Xie H, Turner T, Wang M-H, Singh RK, Siegal GP and Wells A (1995) In vitro invasiveness of DU145 human prostate carcinoma cells is modulated by EGF receptor-mediated signals. *Clin Exp Metastasis* **13**: 407–419
Zhau HY, Chang S-M, Chen B-Q, Wang Y, Zhang H, Kao C, Sang QA, Pathak SJ and Chung LW (1996) Androgen-repressed phenotype in prostate cancer. *Proc Natl Acad Sci USA* **93**: 15152–15157

*© Cancer Research Campaign 1999*
